# Supplementary material for: Presenilin-1 Familial Alzheimer Mutations Impair γ-Secretase Cleavage of APP Through Stabilized Enzyme–Substrate Complex Formation
Source: Biomolecules. 2025 Jul 1;15(7):955. doi: 10.3390/biom15070955 (PMC12292344; doi:10.3390/biom15070955)
Supplement: Supplementary file 1 [file biomolecules-15-00955-s001.zip › biomolecules-3674406-supplementary.pdf]

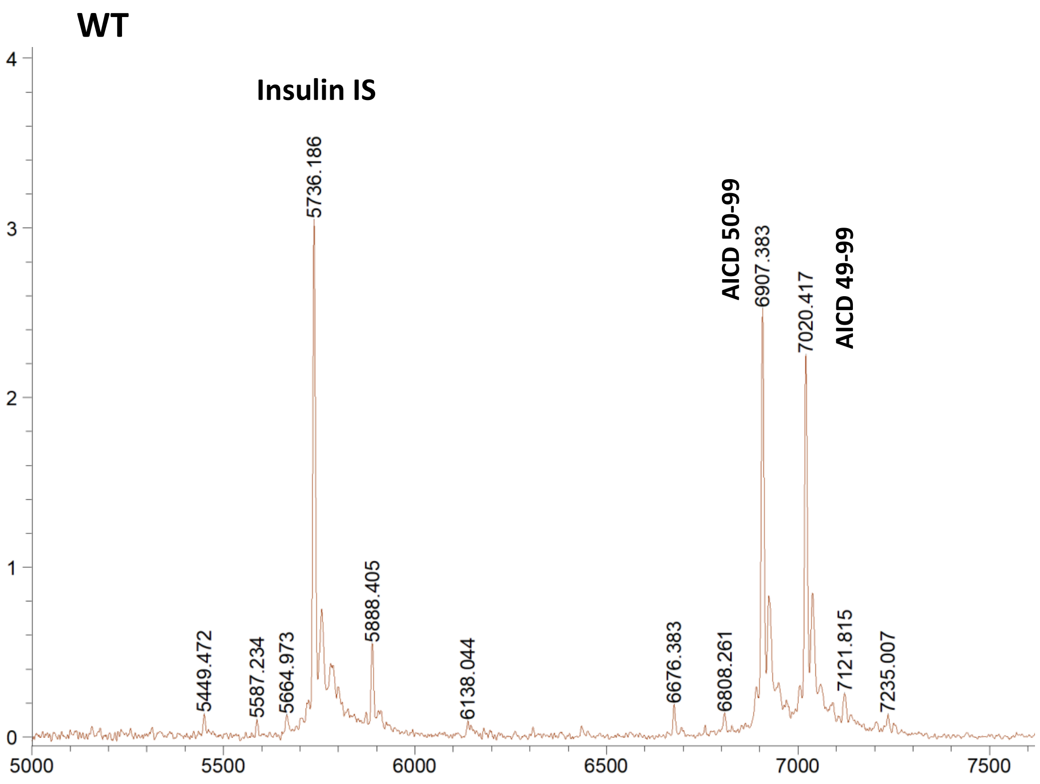

WT
Insulin IS
AICD 50-99
AICD 49-99
5449.472
5587.234
5664.973
5736.186
5888.405
6138.044
6676.383
6808.261
6907.383
7020.417
7121.815
7235.007
0
1
2
3
4
5000
5500
6000
6500
7000
7500

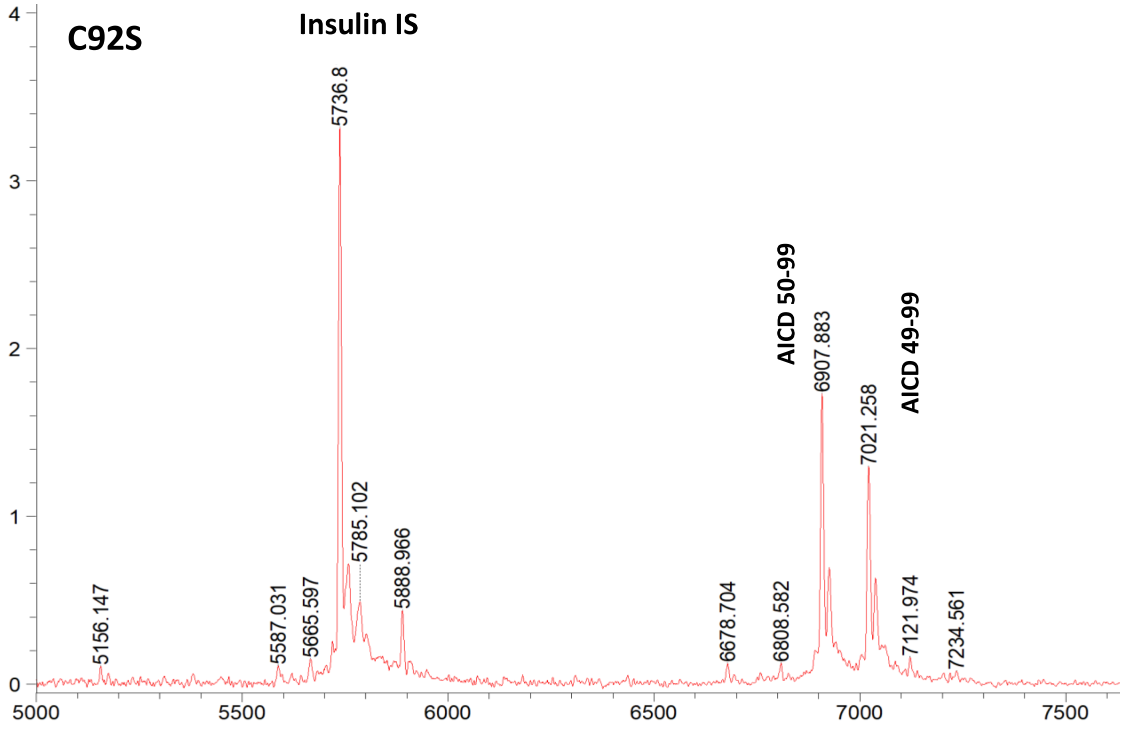

C92S
Insulin IS
AICD 50-99
AICD 49-99
5156.147
5587.031
5665.597
5736.8
5785.102
5888.966
6678.704
6808.582
6907.883
7021.258
7121.974
7234.561
0
1
2
3
4
5000
5500
6000
6500
7000
7500

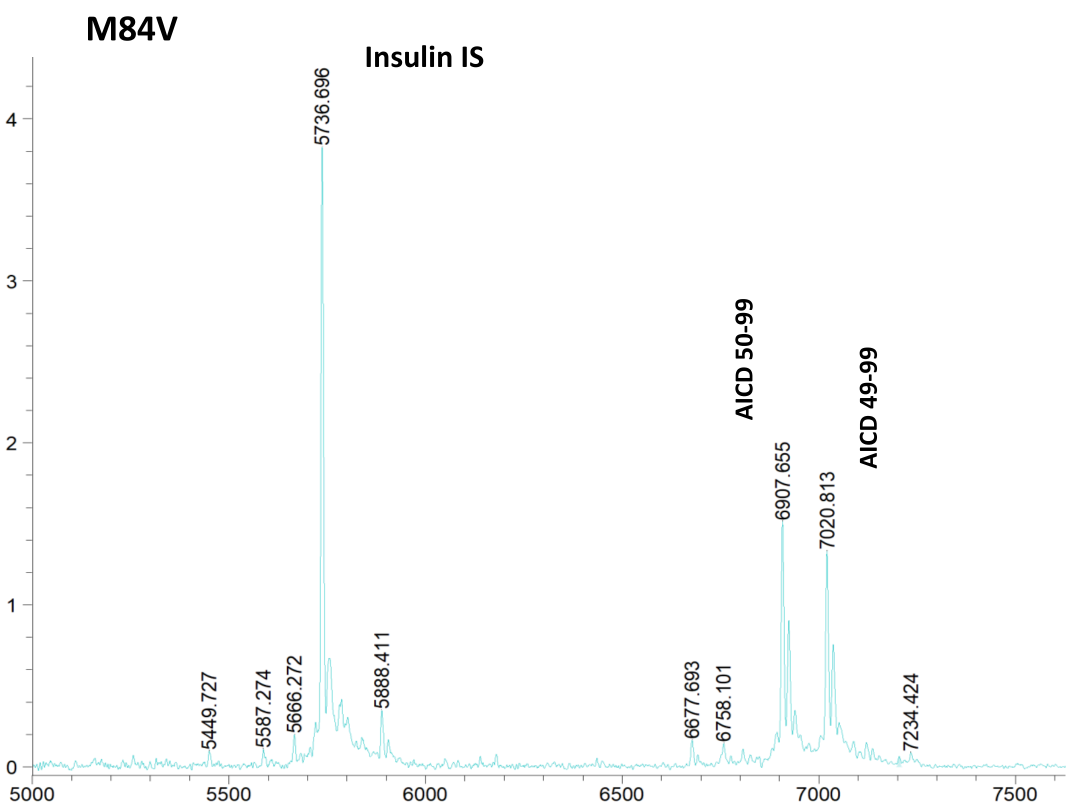

M84V
Insulin IS
AICD 50-99
AICD 49-99
5736.696
6907.655
7020.813
5449.727
5587.274
5666.272
5888.411
6677.693
6758.101
7234.424

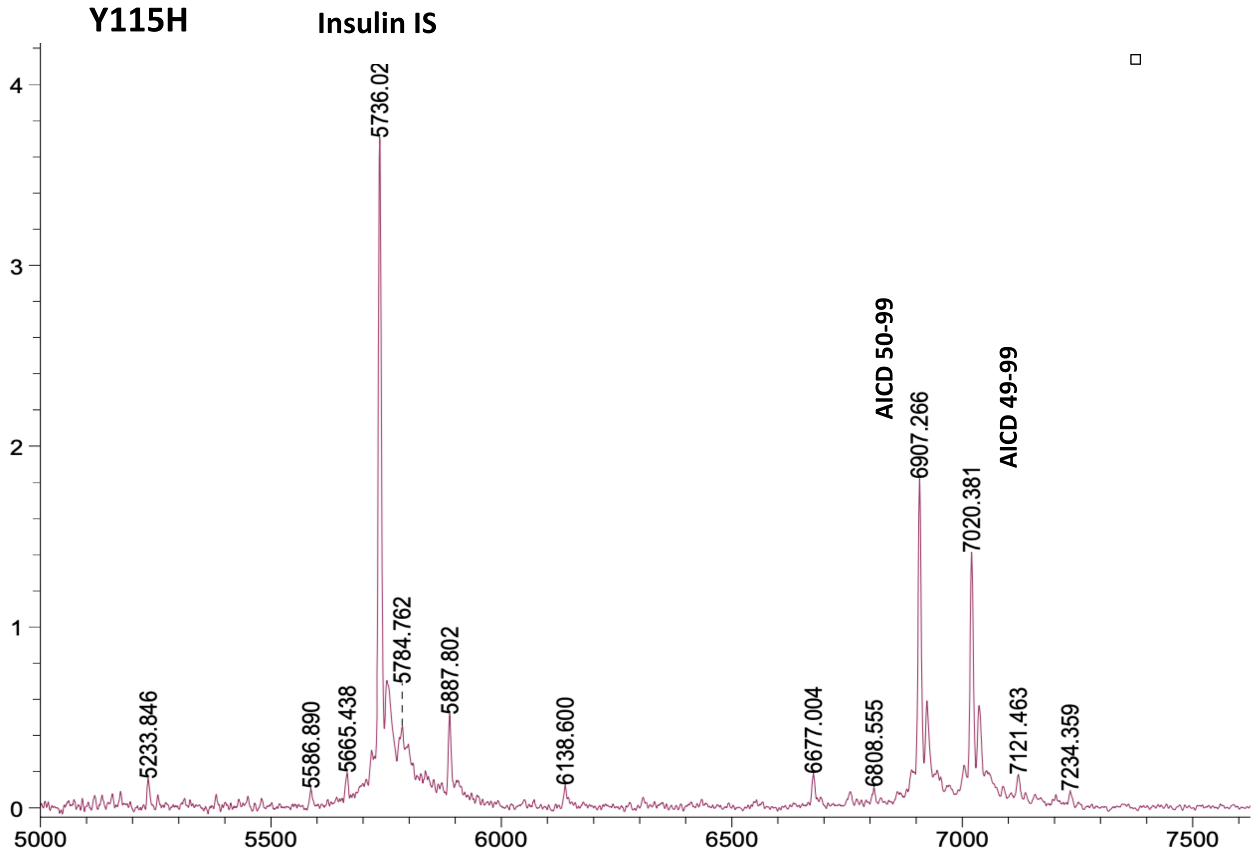

Y115H
Insulin IS
AICD 50-99
AICD 49-99
5736.02
6907.266
7020.381
5233.846
5586.890
5665.438
5784.762
5887.802
6138.600
6677.004
6808.555
7121.463
7234.359

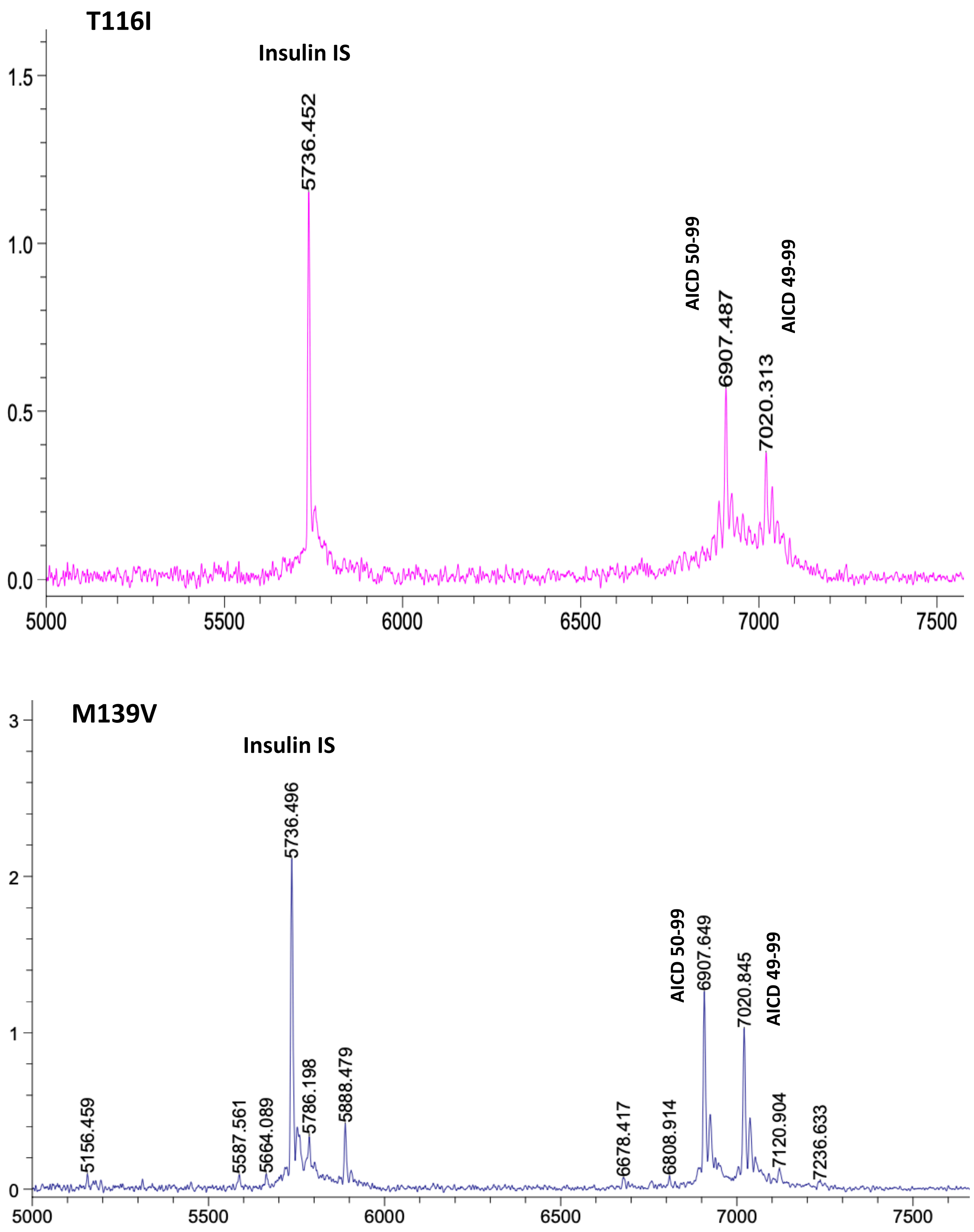

Figure S1. MALDI-TOF Mass detection of (AICD) 50–99 and AICD 49–99 products from wild-type (WT) and six PSEN1 familial Alzheimer's disease (FAD)-mutant γ-secretase along with internal standard.

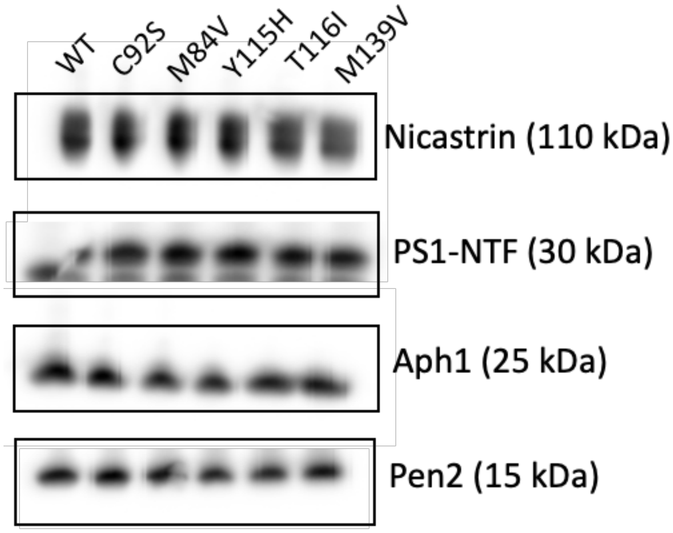

Figure S2. Western blot analysis of all components of purified wild-type (WT) and familial Alzheimer's disease (FAD)-mutant γ-secretase complexes.
